# Comparative performance of IDEXX SDMA Test and the DLD SDMA ELISA for the measurement of SDMA in canine and feline serum

**DOI:** 10.1371/journal.pone.0205030

**Published:** 2018-10-15

**Authors:** Rie Ernst, Jennifer Ogeer, Donald McCrann, Julie Cross, Marilyn Strong-Townsend, Hanne Friis, Michael Coyne, Celeste Clements, Corie Drake, Rachel Murphy

**Affiliations:** 1 Fredrikstad Dyrehospital, Fredrikstad, Norway; 2 IDEXX Laboratories, Inc., Westbrook, Maine, United States of America; 3 IDEXX Europe B.V. Hoofddorp, The Netherlands; Colorado State University, UNITED STATES

## Abstract

Kidney disease is common in companion animals, and traditionally diagnosed with serum creatinine concentration (sCr), blood urea nitrogen, and abnormal urinalysis findings. Symmetric dimethylarginine (SDMA) is a novel kidney biomarker that reflects glomerular filtration rate, increasing earlier than sCr with acute kidney injury and chronic kidney disease. This prospective study compared accuracy and precision of two commercial SDMA assays, the IDEXX SDMA Test and the DLD SDMA ELISA, relative to the established reference method, liquid chromatography/mass spectrometry (LC-MS). Thirty canine and 30 feline pooled serum samples were used to evaluate accuracy compared to LC-MS. Pooled canine samples with a low SDMA concentration and pooled feline samples with a high SDMA concentration were used to evaluate precision. Using a best fit linear model, the IDEXX SDMA Test resulted in a slope of 1.06 and an intercept of 0.34, with R^2^ = 0.99, and the DLD SDMA ELISA resulted in a slope of 0.37 and an intercept of 11.33, with R^2^ = 0.27, when compared to LC-MS. Estimated bias over a clinically relevant range for SDMA (10–45 μg/dL) was 1–2 μg/dL for the IDEXX SDMA Test, while DLD SDMA ELISA showed considerable bias, 5–8 μg/dL. Day-to-day precision analysis of the low SDMA concentration samples showed 7.7% total coefficient of variation (CV) for the IDEXX SDMA Test and 31.1% for the DLD SDMA ELISA. For the high SDMA concentration samples, total CV was 2.3% for the IDEXX SDMA Test and 28.2% for the DLD SDMA ELISA. In this study the IDEXX SDMA Test was more accurate and more precise in macroscopically normal serum than the DLD SDMA ELISA when compared to the reference method of LC-MS. The IDEXX SDMA Test is more suitable for clinical use in the diagnosis and monitoring of kidney disease in dogs and cats.

## Introduction

Kidney disease is common in small animals, with chronic kidney disease (CKD) recognized as an important cause of morbidity and mortality in cats [1, 2] and dogs [3]. Early diagnosis and nutritional management of kidney disease are recommended to slow progression and improve survival time of cats [2, 4, 5] and dogs [6] diagnosed with CKD. Early decreases in glomerular filtration rate (GFR) are not readily recognized by the commonly used diagnostic tests, including sCr and blood urea nitrogen (BUN) [7–9]. Symmetric dimethylarginine (SDMA) is a methylated form of arginine found within all nucleated cells that is released into circulation after proteolysis, then excreted through the kidneys, and correlates well with GFR in people [10], dogs [3,11], and cats [12–13]. SDMA has been shown to increase earlier than sCr in cats [13] and dogs with CKD [3] and studies have demonstrated that SDMA increases when there is 25%- 40% decrease in GFR [3,11,13]. SDMA is also more specific than sCr, being less impacted by extrarenal factors including body condition and advanced age [14–17]. Since SDMA is not affected by lean body mass [14,15] it is potentially more reliable for assessing kidney function in animals with conditions that result in muscle loss, such as feline hyperthyroidism or advanced CKD. In 2015 IRIS amended the CKD guidelines to incorporate SDMA, along with sCr, for the diagnosis and treatment of CKD in dogs and cats, noting that SDMA may be a more sensitive indicator of kidney function than sCr [18], and that SDMA may be used as an adjunct to sCr to guide treatment for patients with low body-condition scores, in which sCr may underestimate the degree of renal dysfunction [18].

Liquid chromatography- mass spectrometry (LC-MS) analysis for SDMA is considered the gold standard due to its accuracy and precision, but is costly, time-consuming, and is not readily available [19]. Veterinary clinicians need a cost-effective, timely, and accurate test to maintain SDMA as an essential part of a routine chemistry profile. The objective of this prospective study was to evaluate and compare the accuracy and precision of two commercially available SDMA assays, the IDEXX SDMA Test and DLD SDMA ELISA, in cats and dogs, relative to LC-MS, established as the reference method. The IDEXX SDMA Test is a novel, high-throughput, competitive, homogeneous immunoassay for SDMA that was validated on serum and plasma from cats and dogs, both in healthy and CKD populations, according to CLSI standards [20, 21] The SDMA Microtiter Plate ELISA test manufactured by DLD Diagnostika GMBH is designed for measuring SDMA in human samples, and is being offered by some veterinary laboratories.

## Materials and methods

### Sample selection and preparation

Individual serum samples from 209 dogs and from 234 cats were used in the study. Each sample was obtained and submitted to an IDEXX commercial reference laboratory by a practicing veterinarian during the normal diagnostic workup and monitoring of clinically well and ill dogs and cats in his or her care. All samples were obtained on the consent of the pet owner. After submission and analysis, ownership of the samples transferred to IDEXX as per terms of the service contract. The study was approved by the IDEXX Laboratories Animal Welfare Review Committee and complies with its guidelines.

Samples were required to have SDMA and SCr levels determined, a minimum serum volume of 0.5 mL, and minimal lipemia, icterus or hemolysis. SDMA was determined using a commercially available high-throughput immunoassay (IDEXX SDMA Test; IDEXX Laboratories Inc., One IDEXX Drive, Westbrook, Maine 04092, USA). SCr were determined by a colorimetric method, Jaffe’s reaction using picrate at alkaline pH [22] (Beckman Coulter, Inc, Brea CA). Samples submitted to an IDEXX commercial laboratory that met the above conditions were then stratified by SDMA concentration into 4 ranges (0–14 μg/dL, 15–25 μg/dL, 26–50 μg/dL, and > 50 μg/dL) and randomly selected from within each range. This was to ensure that selected samples had SDMA concentrations that spanned a wide analytic range. Selected samples were stored for a maximum of 7 days. Each sample was identified by the breed, age, and sex of the dog or cat from which the sample was obtained. To ensure privacy, demographic information on the pet, pet owner, or veterinarian who submitted the sample was not collected.

Once weekly, all samples were sent to a central facility (IDEXX Laboratories, Inc., Westbrook, ME) for study preparation. Samples were stored at -20°C for a maximum of 21 days prior to preparation. Using the IDEXX SDMA Test measurements from the reference laboratory, individual canine samples and feline serum samples were combined to create 35 canine and 35 feline pooled serum samples containing a minimum of 2.0 mL. Between 2 to 4 individual serum samples were combined for each pooled serum sample. The SDMA concentration in each pooled serum sample was determined using LC-MS following previously described methods [11]. The mean value of 3 LC-MS determinations per sample was used. Thirty canine pooled serum samples and 30 feline pooled serum samples were then selected based on the LC-MS measured SDMA concentration to ensure that the selected samples spanned a wide dynamic range. Paired samples were aliquoted, labeled with the same unique identification and frozen at -80°C.

Additional individual canine serum samples were pooled to obtain a bulk sample with low SDMA concentration (target: 10 to 15 μg/dL) and individual feline serum samples were pooled to obtain a bulk sample with high SDMA concentration (target: 30 to 35 μg/dL). To reach the target concentration for the high SDMA bulk sample, exogenous SDMA was added. The SDMA concentration in each bulk serum sample was determined using the mean of 3 LC-MS determinations following standard techniques noted previously. Sixty samples of each bulk serum sample were aliquoted, labeled with a unique identification, and frozen at -80°C.

One set of each of the individual canine accuracy samples (n = 30), the feline accuracy samples (n = 30), the pooled canine low SDMA samples (n = 30) and the pooled feline high SDMA samples (n = 30) were shipped frozen to the Fredrikstad Dyrehospital veterinary facility using overnight international shipping. Eight samples (2 canine accuracy, 2 feline accuracy, 2 canine low precision, 2 feline high precision) were randomly selected and submitted daily over 15 days to a Laboklin commercial laboratory (Laboklin GmbH&Co. KG, Steubenstraße 4, D—97688 Bad Kissingen, Germany) for evaluation of SDMA and sCr concentrations. Measurement of sCr was performed to ensure the clinical integrity and identity of the analyzed samples that were submitted masked to the two laboratories. SDMA was determined by a commercial competitive ELISA method using the microtiter plate format, DLD SDMA ELISA, (DLD Diagnostika GmbH, Adlerhorst 15, D-22459 Hamburg, Germany) and reported as mmol/L. SCr concentrations were determined by a colorimetric method (Jaffe Gen.2, Roche Diagnostics) and reported as μmol/L. Samples were thawed and shipped on ice Monday through Thursday of each week to the commercial laboratory using standard overnight delivery methods. Samples were masked so that only the species from which the sample was obtained was provided to the laboratory.

The retained, paired samples were submitted to an IDEXX commercial laboratory (IDEXX Laboratories, Inc. 52 Church Hill Rd, Newtown, CT 06470) for evaluation of IDEXX SDMA Test and sCr concentrations. Eight samples were submitted over 15 days following the same submission order as used previously. SCr concentrations were determined by the Jaffe reaction as previously described and reported as mg/dL.

Samples were processed and evaluated by the Laboklin and IDEXX commercial laboratories within 3 months and 5 months of the date of sample collection, respectively.

### Statistical analysis

Prior to analysis, SDMA and sCr concentrations obtained from the Laboklin Laboratory were converted using standard unit conversion from mmol/L to μg/dL and μmol/L to mg/dL, respectively. To evaluate assay accuracy, difference plots and ordinary linear regression were used to examine bias and correlation to the reference standard. To evaluate assay precision, standard deviations for total, within, and between day precision were estimated using restricted maximum likelihood (REML) variance components analysis. Outliers were removed to ensure the data sets correctly represented the study population, using the extreme studentized deviate (ESD) test [23]. SCr of samples were compared between laboratories using scatter plot and rank correlation.

## Results

### Accuracy

**Fig 1:**
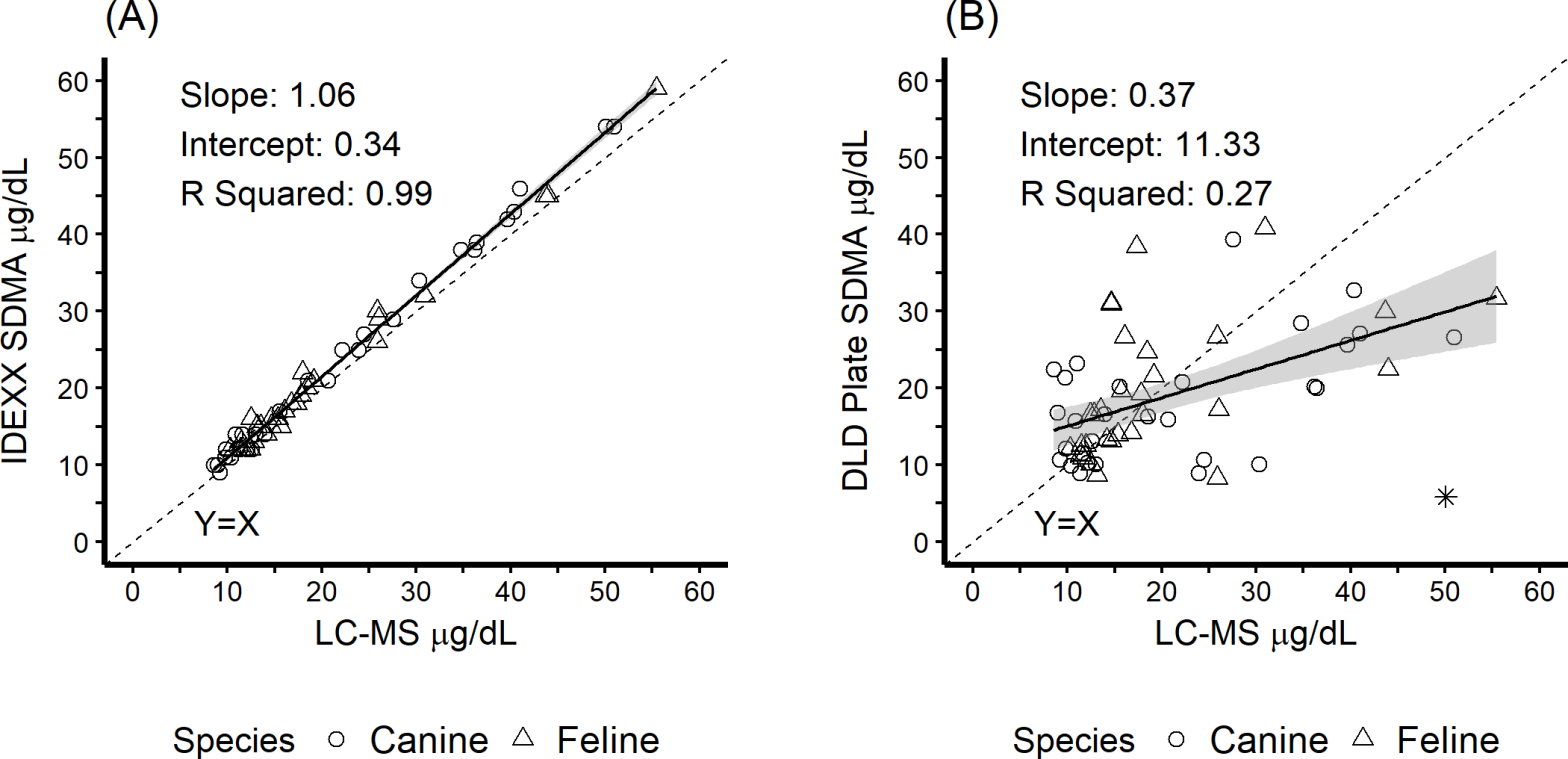
Accuracy of two SDMA diagnostic methods compared to the reference standard (LC-MS). (A) The IDEXX SDMA Test. (B) The DLD SDMA ELISA method. Solid line represents best fit linear model; shading represents 95% confidence interval around the line. Dashed line is the line of equality. Outliers are represented by *.

The ranges of SDMA concentrations measured by LC-MS for the 30 individual canine and 30 individual feline samples were 9–51 μg/dL and 10–55 μg/dL, respectively. The accuracy of the two SDMA diagnostic methods is illustrated in Fig 1. Outlier analysis resulted in the removal of one measure in the DLD SDMA ELISA group, as shown in Fig 1(B). The best fit linear model for the IDEXX SDMA Test resulted in a slope of 1.06 (95% CI: 1.03, 1.08), an intercept of 0.34 (95% CI: -0.25, 0.92) with R^2^ = 0.99. The residual standard error was 1.13. The best fit linear model for the DLD SDMA ELISA resulted in a slope of 0.37 (95% CI: 0.21, 0.53), an intercept of 11.33 (95% CI: 7.52, 15.14) with R^2^ = 0.27. The residual standard error was 7.19. Regression analysis confirmed that the species from which the serum was obtained was not significant factor for either the IDEXX SDMA Test or the DLD SDMA ELISA method. For clarity, slopes and intercepts with 95% confidence intervals for the best fit linear models were determined for both canine and feline samples individually as shown in Table 1.

**Table 1:** Slopes and intercepts with 95% confidence intervals for the best fit linear models determined for all samples and for canine and feline samples individually.

Model	Test	Species	Slope (95% CI)	Intercept (95% CI)	R^2^
Accuracy	IDEXX SDMA Test	Overall	1.06 (1.03, 1.08)	0.34 (-0.25, 0.92)	0.99
		Canine	1.07 (1.04, 1.10)	0.33 (-0.42, 1.07)	
		Feline	1.04 (1.00, 1.08)	0.50 (-0.44, 1.45)	
	DLD SDMA ELISA	Overall	0.37 (0.21, 0.53)	11.33 (7.52, 15.14)	0.27
		Canine	0.36 (0.15, 0.56)	10.52 (5.51, 15.53)	
		Feline	0.40 (0.13, 0.68)	11.76 (5.68, 17.84)	
Bias	IDEXX SDMA Test	Overall	0.06 (0.03, 0.08)	0.34 (-0.25, 0.92)	N/A
		Canine	0.07 (0.04, 0.10)	0.33 (-0.42, 1.07)	
		Feline	0.04 (0.00, 0.08)	0.50 (-0.44, 1.45)	
	DLD SDMA ELISA	Overall	-0.63 (-.079, -0.47)	11.33 (7.52, 15.14)	N/A
		Canine	-0.64 (-0.85, -0.44)	10.52 (5.51, 15.53)	
		Feline	-0.60 (-0.87, -0.32)	11.76 (5.68, 17.84)	

N/A = not applicable.

**Fig 2:**
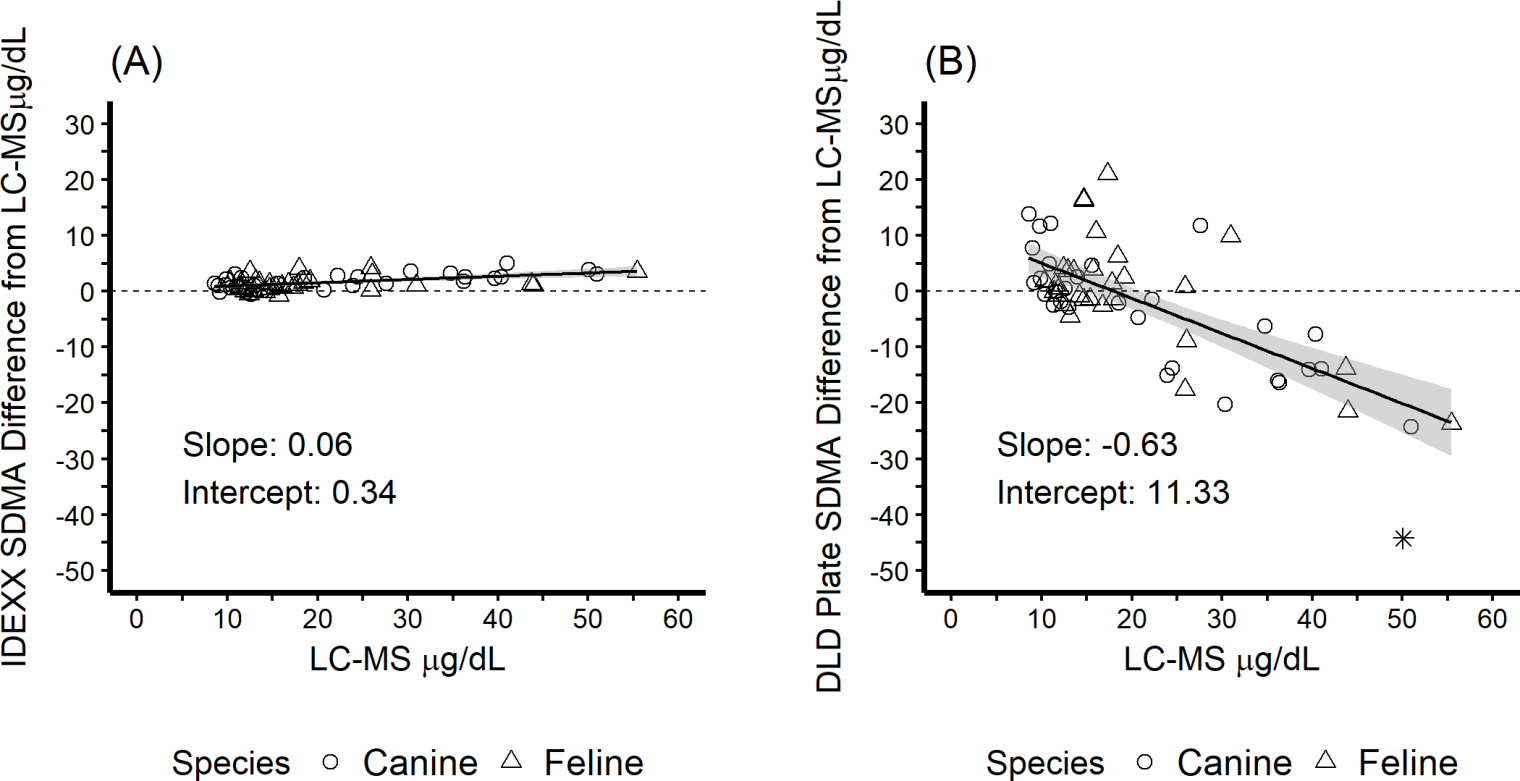
Bias of two SDMA diagnostic methods–difference from the reference standard (LC-MS). (A) The IDEXX SDMA Test. (B) The DLD SDMA ELISA method. Solid line represents best fit linear model; shading represents 95% confidence interval around the line. Dashed line is the line of equality. Outliers are represented by *.

The bias of the two SDMA diagnostic methods is illustrated in Fig 2. The best fit linear model for the IDEXX SDMA Test resulted in a slope of 0.06 (95% CI: 0.03, 0.08) and an intercept of 0.34 (95% CI: -0.25, 0.92). The best fit linear model for the DLD SDMA ELISA resulted in a slope of -0.63 (95% CI: -0.79, -0.47) and an intercept of 11.33 (95% CI: 7.52, 15.14). For clarity, slopes and intercepts with 95% confidence intervals for the best fit linear models were determined for both canine and feline samples individually as shown in Table 1. Minimal bias was noted for the IDEXX SDMA Test, as compared to the DLD SDMA ELISA (Table 2).

**Table 2:** Systematic bias calculated from fit lines of IDEXX SDMA Test and DLD ELISA SDMA compared to LC-MS over a clinically relevant range of SDMA concentrations.

	Systematic Bias (μg/dL)	
SDMA Value LC-MS	IDEXX SDMA Test	DLD ELISA SDMA
10 μg/dL	0.94	5.03
14 μg/dL	1.18	2.51
20 μg/dL	1.54	-1.27
30 μg/dL	2.14	-7.57
45 μg/dL	3.04	-17.02

Negative numbers indicate the assay is under recovering SDMA with respect to the reference (LC-MS)

**Fig 3:**
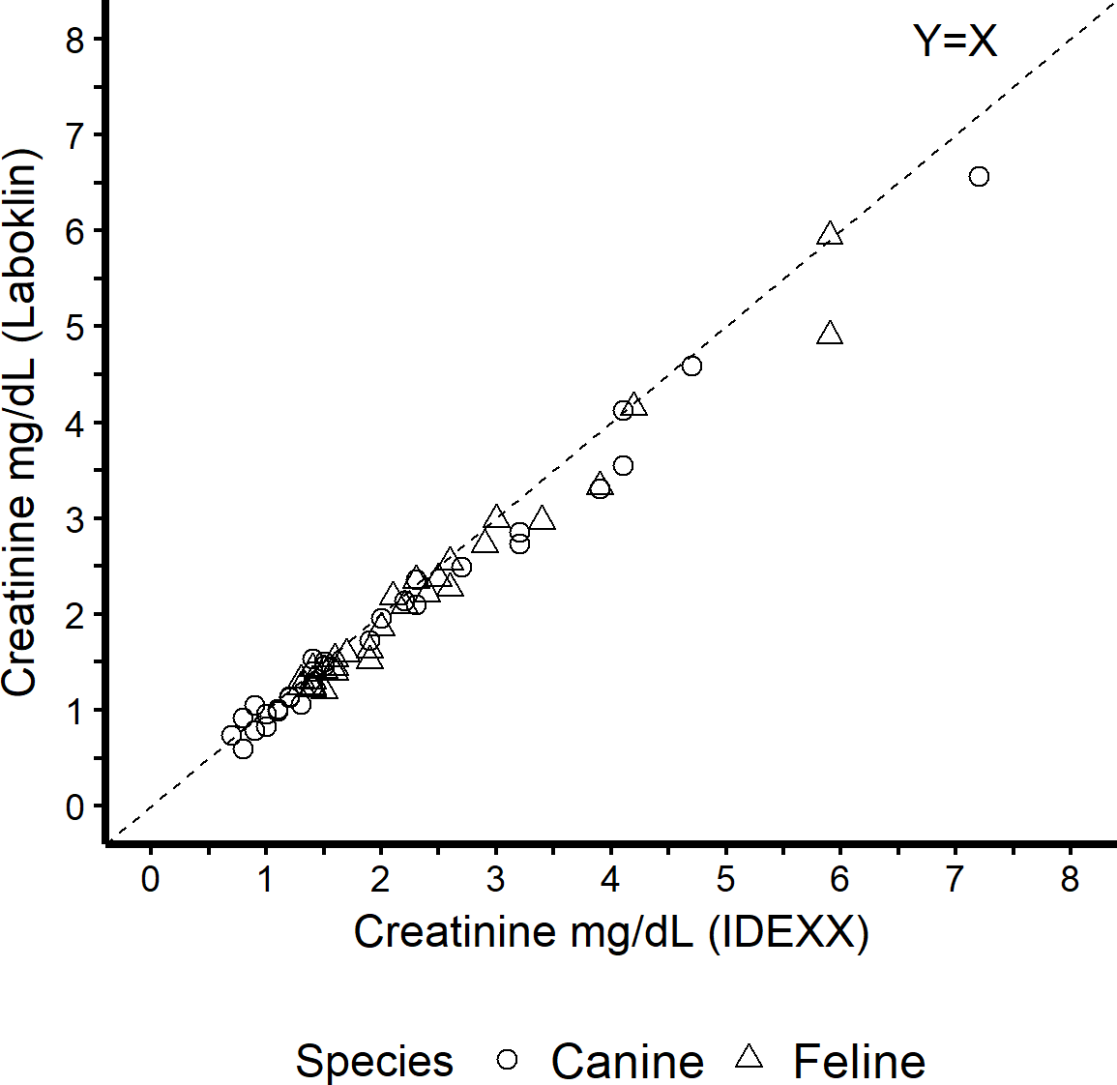
Comparison of creatinine (sCr) measures between laboratories. Dashed line is the line of equality.

The comparison of sCr between laboratories is show in Fig 3. Strong correlation was seen between the sCr results from both Laboklin and IDEXX laboratories (Spearman’s rank correlation, ρ = 0.96).

### Precision

**Fig 4:**
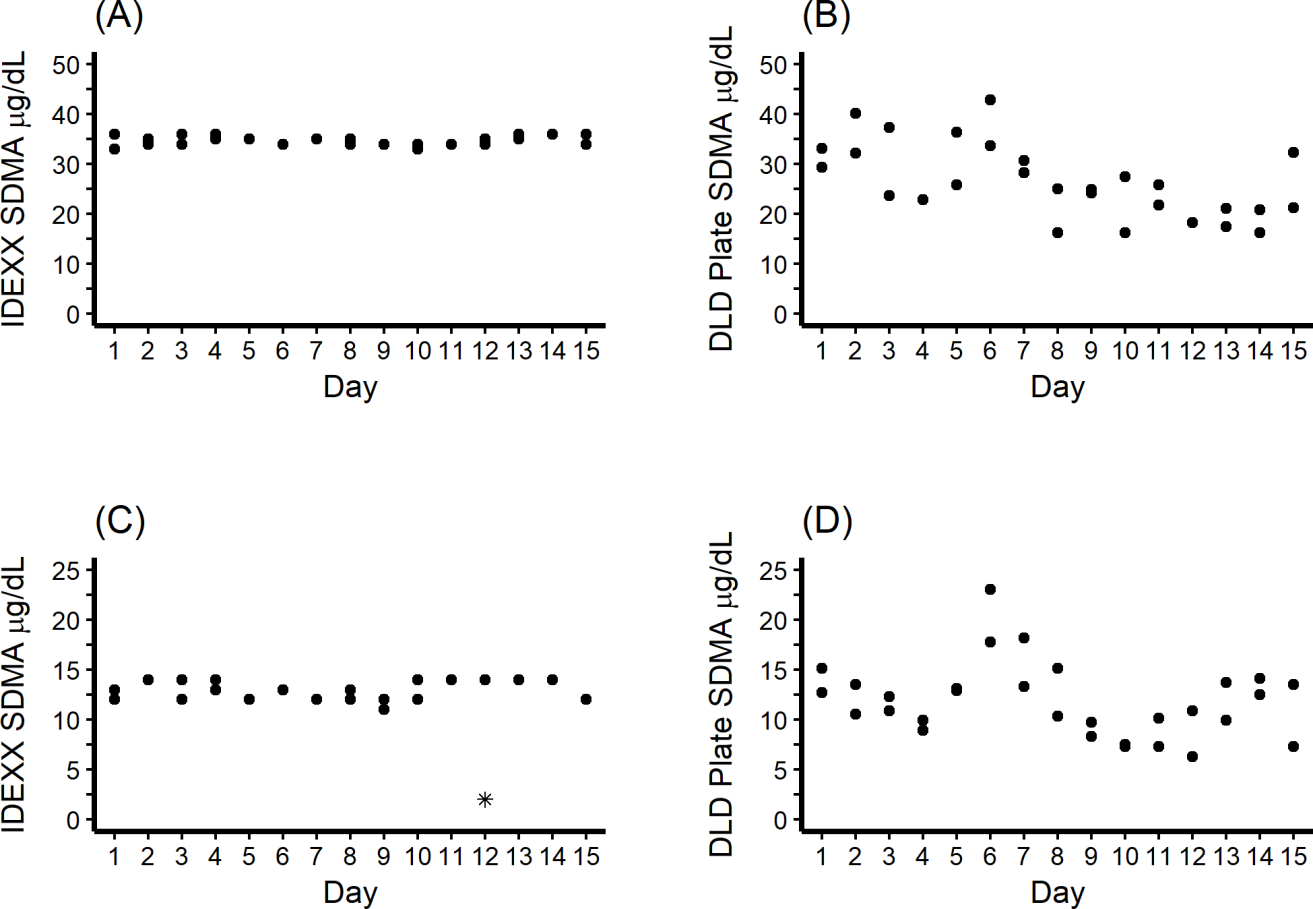
Day-to-day precision of two SDMA diagnostic methods compared to the reference standard (LC-MS). (A) The IDEXX SDMA Test–Feline sample, 31.6 μg/dL (LC-MS). (B) The DLD SDMA ELISA method–Feline sample, 31.6 μg/dL (LC-MS). (C) The IDEXX SDMA Test–Canine sample, 11.8 μg/dL (LC-MS). (D) The DLD SDMA ELISA method–Canine sample, 11.8 μg/dL (LC-MS). Outliers are represented by *.

The SDMA concentration in the pooled serum samples was measured by LC-MS and was determined to be 11.8 μg/dL (target: 10 to 15 μg/dL) for the pooled canine serum sample and 31.6 μg/dL (target: 30 to 35 μg/dL) for the pooled feline serum sample. The day-to-day precision of the two SDMA diagnostic methods is show in Fig 4. Outlier analysis resulted in the removal of one measure in the IDEXX SDMA Test group, as shown in Fig 4(C).

The total variation by assay for each pooled serum sample, along with the within- and between-day assay variation and total coefficient of variation are found in Table 3.

**Table 3:** Variation by assay for each pooled serum sample.

Method	Species	Mean (μg/dL)	Coefficient of Variation Total	Standard Deviation Total (95% CI)	Standard Deviation Within-Day (95% CI)	Standard Deviation Between-Day (95% CI)
IDEXX SDMA Test Low	Canine	13.0	7.7%	*1.0 (0.8, 1.4)	0.7 (0.5, 1.0)	0.7 (0, 1.1)
DLD SDMA ELISA Low	Canine	11.9	31.1%	3.7 (2.9, 5.3)	2.5 (1.8, 3.8)	2.8 (0, 4.1)
IDEXX SDMA Test High	Feline	34.7	2.3%	0.8 (0.5, 1.5)	0.8 (0.4, 1.8)	0.1 (0, 0.5)
DLD SDMA ELISA High	Feline	26.2	28.2%	7.4 (5.7, 10.4)	5.3 (3.9, 8.2)	5.1 (0, 7.6)

*Total SD including outlier = 2.28 (1.8, 3.0)

SDMA and sCr sample measures for each laboratory are found in S1 Dataset.

## Discussion

The diagnosis of kidney disease is multifaceted, including assessment of patient signalment, history, clinical signs, physical examination findings, and results of diagnostic testing, including hematology, biochemistry, urinalysis, medical imaging and ancillary evaluations such as blood pressure measurement. Veterinary clinicians utilize laboratory assessment of sCr and BUN to diagnose kidney disease, analytes which have been shown to be insensitive, late markers of kidney dysfunction [7–9, 22] and non-specific, namely, influenced by extrarenal factors [22]. SDMA is a more sensitive and earlier indicator of kidney function that can indicate progressive kidney function loss before other parameters and is less influenced by extra-renal factors [3, 11, 13–15]. Accurate and precise SDMA measurements are needed to optimize patient diagnosis and management based on standardized IRIS CKD staging and individual patient concerns. When compared to SDMA measured by LC-MS on the same individual patient samples, IDEXX SDMA Test results showed strong agreement. In this study SDMA concentrations measured with the IDEXX SDMA Test were substantially more accurate and more precise in macroscopically normal serum than those measured with the DLD SDMA ELISA when compared to the reference method of LC-MS.

Bias of a diagnostic test can negatively impact appropriate medical interpretation. For the IDEXX SDMA Test estimated bias over the clinically relevant range (10–45 μg/dL) was 1 to 3 μg/dL. In contrast, the DLD SDMA ELISA showed considerable bias over the clinically relevant range, from 5 to -17 μg/dL. For the IDEXX SDMA Test a small proportional bias was noted for canine and feline samples near the cutoff of 14 μg/dL, (Fig 2(A)); whereas, the DLD SDMA ELISA revealed a moderate bias (Fig 2(B)). The biases reported for the DLD SDMA ELISA not only impact the accurate assessment of kidney function in individual diagnostic tests, but also alter the therapeutic recommendations and laboratory monitoring of patients for progressive kidney disease. Near the SDMA cutoff of 14 μg/dL the DLD SDMA ELISA can overestimate or underestimate kidney function, which could lead to a false positive diagnosis of kidney disease, or perhaps more importantly, a failure to diagnose early kidney disease. Missed opportunities to identify and treat reversible causes and contributors to progressive kidney disease may negatively impact patient outcome. At concentrations of SDMA ≥ 30 μg/dL the DLD SDMA ELISA had a negative bias of ≥ 7.57 μg/dL (Table 2), which could lead to incorrect assessment of moderate to severe kidney disease, as kidney function is overestimated. The difference in systematic bias between the two methods supports the clinical use of the more accurate IDEXX SDMA Test rather than DLD SDMA ELISA.

Using a reliable, precise SDMA assay for patient monitoring is essential to recognize clinically significant differences between consecutive laboratory tests. For any analyte, imprecision, that is, specific assay variation, combined with biological variation, contributes to the expected variation of a given result that occurs independent of disease or dysfunction. The level of imprecision that is acceptable can vary throughout the assay range, and can depend on the clinical utility of the analyte. When calculating the analytical/functional sensitivity of an immunoassay, at some point, as the analyte concentration decreases, the imprecision increases and the signal is essentially lost in the noise of the assay. This point, referred to as the lower limit of quantitation, is often set at an imprecision (Coefficient of Variation) of < 20%, and is regarded as the absolute highest imprecision that would be tolerated in a meaningful immunoassay result [24]. At higher analyte concentrations, imprecision of less than 10% would be ideal [25].

The IDEXX SDMA Test showed excellent precision day- to- day and across the 15- individual- day period for measurements of a single, pooled high SDMA sample (Fig 4(A)) and low SDMA sample (Fig 4(C)) determined by LC-MS as the reference standard. As shown in Table 3 total standard deviation for the pooled samples was estimated as ≤ 1.0 μg/dL. By contrast DLD SDMA ELISA measurements were imprecise within days and across 15 days of time (Fig 4(B) and 4(D)), and total standard deviation was estimated as 3.7 μg/dL for low SDMA and 7.4 μg/ for high SDMA concentrations (Table 3). In the laboratory evaluation of kidney function a high degree of imprecision could result in a failure to diagnose kidney disease, either acute or chronic, or to recognize disease progression. Either might result in more poor patient outcomes from lack of appropriate medical intervention. With an imprecise SDMA measurement that is falsely increased, over-diagnosis of kidney disease or disease progression could lead to unnecessary patient testing or hospitalization that also causes unwarranted expense and owner concern. This could result in loss of owner confidence in the veterinarian’s decision-making and could interfere with owner compliance and appropriate patient care.

Data in Fig 3 show good correlation between paired sCr measured concurrent with IDEXX SDMA Test and DLD SDMA ELISA at their corresponding commercial laboratories. The purpose of evaluating serum creatinine at each laboratory was to ensure the clinical integrity and identity of the analyzed samples that were submitted masked to the Laboklin and IDEXX laboratories and not to compare analytic methodologies. Although a reference method was not used, the strong correlation between methods supports that proper sample handling/labeling was used throughout the study. If the disagreement between SDMA methods were the result of sample mishandling/mislabeling, we would not expect to see such strong correlation of serum creatinine.

Despite careful attention to design there were some limitations inherent in this study. Samples were analyzed routinely in blinded fashion at two commercial laboratories rather than side-by-side. While IDEXX SDMA testing was performed on a single instrument with standard quality control guidelines, the authors had no control over laboratory specimen processing or instrument control for analysis of the DLD SDMA ELISA at Laboklin laboratory; procedural information was limited to that provided in the manufacturer’s package insert. This study does not compare analytical performance with common sample interferences such as lipemia, icterus, or hemolysis that are encountered routinely in clinical practice as only macroscopically normal samples were used. Finally, all serum samples were frozen prior to testing. Previous studies have shown that dog serum SDMA concentrations remain stable for 7 days at room temperature, 20°C and for 14 days at 4°C, with insignificant loss after exposure to 3 freeze-thaw cycles [11]. To the authors’ knowledge there are no published data on the effects of storage or freeze-thaw cycles on cat SDMA concentrations, or long-term storage of dog samples. Such studies could be performed at a future date; however, the current study was designed to minimize sample handling differences during processing and reanalysis that might affect sample integrity: all samples compared to SDMA LC-MS were handled in similar fashion.

## Conclusions

In this study SDMA concentrations measured with the IDEXX SDMA Test were more accurate and more precise in macroscopically normal serum than those measured with the DLD SDMA ELISA when compared to the reference method of LC-MS. The IDEXX SDMA Test is more suitable for clinical use in the diagnosis and monitoring of kidney disease in dogs and cats.

## Supporting information

S1 Dataset SDMA and SCr results from both Laboklin and IDEXX Laboratories. (CSV)
